# Polyp Detection from Colorectum Images by Using Attentive YOLOv5

**DOI:** 10.3390/diagnostics11122264

**Published:** 2021-12-03

**Authors:** Jingjing Wan, Bolun Chen, Yongtao Yu

**Affiliations:** 1 Department of Gastroenterology, The Affiliated Huai’an Hospital of Xuzhou Medical University, The Second People’s Hospital of Huai’an, Huaian 223002, China; 11000419@hyit.edu.cn; 2 Department of Computer Science, Huaiyin Institute of Technology, Huaiyin 223001, China; allennessy@hyit.edu.cn; 3 Department of Physics, University of Fribourg, CH-1700 Fribourg, Switzerland

**Keywords:** colorectal cancer, polyp detection, YOLOv5, attention mechanism

## Abstract

Background: High-quality colonoscopy is essential to prevent the occurrence of colorectal cancers. The data of colonoscopy are mainly stored in the form of images. Therefore, artificial intelligence-assisted colonoscopy based on medical images is not only a research hotspot, but also one of the effective auxiliary means to improve the detection rate of adenomas. This research has become the focus of medical institutions and scientific research departments and has important clinical and scientific research value. Methods: In this paper, we propose a YOLOv5 model based on a self-attention mechanism for polyp target detection. This method uses the idea of regression, using the entire image as the input of the network and directly returning the target frame of this position in multiple positions of the image. In the feature extraction process, an attention mechanism is added to enhance the contribution of information-rich feature channels and weaken the interference of useless channels; Results: The experimental results show that the method can accurately identify polyp images, especially for the small polyps and the polyps with inconspicuous contrasts, and the detection speed is greatly improved compared with the comparison algorithm. Conclusions: This study will be of great help in reducing the missed diagnosis of clinicians during endoscopy and treatment, and it is also of great significance to the development of clinicians’ clinical work.

## 1. Introduction

In China, the incidence rate of colorectal cancer (CRC) has been increasing year by year. It has jumped to the top 3–5 of cancers with the greatest mortality. Colorectal cancer is the third and second largest cause of cancer-related death in men and women [1]. This is increasingly affecting people’s health and quality of life. According to statistics, by 2020, nearly 150,000 people will have been diagnosed with CRC, and more than 50,000 people will have died of the disease [2]. It must be mentioned that colorectal cancer has the fastest rising cancer incidence rate in recent years. The number of new cases and deaths has doubled in the past 10 years and is increasing at an annual average of 4–5%. In addition, data from epidemiological studies show that the incidence of the CRC in adults under the age of 50 is already significantly high and continues to rise [3]. Sedentary, obesity, high protein foods, excessive intake of salted foods and high life pressure are all external causes of colorectal cancer. In view of its high incidence rate and mortality, the prevention of CRC is an urgent problem to be solved. Studies have found that most CRC cases evolved gradually from colorectal polyps, especially adenomatous polyps. Timely resection of polyps can effectively prevent the occurrence of CRC and reduce CRC-related mortality by 70% [4].

Studies have shown that colonoscopy is considered to be the gold standard for reducing the incidence rate and mortality of colorectal cancers [5,6]. Adenoma detection rate (ADR) is the most common prevention for colorectal cancers. On the contrary, if the adenoma is not found in time, it may lead to the occurrence and development of the interphase cancer. However, due to the individual differences in the technical level of endoscopists, the detection rate of adenoma ranges from 7% to 53%. Colonoscopy and adenomatous polyps can reduce the incidence rate and mortality of CRC. It has been reported that CRC mortality can be reduced by more than 50% [7,8,9,10]. In addition, there is evidence that the risk of interphase CRC decreases by 3.0–6.0% for every 1.0% increase in the detection rate of the adenoma [11]. However, due to the characteristics of polyps and the individual differences in the technical level of endoscopists, polyps may be omitted. It has been reported that the polyp omission rate is as high as 27% [12]. Studies have shown that there is a significant correlation between the polyp detection rate (PDR) and the ADR. The PDR can be used as an ADR alternative index for the quality of colonoscopy in patients with gastrointestinal diseases [13]. Therefore, reducing the number of missed adenomas/polyps by some effective means to standardize the quality of colonoscopy is a hot issue in CRC prevention.

At present, the medical industry has integrated more emerging technologies such as artificial intelligence and deep learning. In the field of gastroenterology, these technologies can create an intelligent auxiliary system that can automatically detect and describe polyp information for a large number of videos and imaging data generated in the colorectal screening process, which helps to overcome the limitations of traditional colonoscopy and improve the quality of colonoscopy screening. Therefore, they make medical services intelligent in real sense and promote the prosperity and development of medical undertakings [14,15].

In July 2021, Professor Bernal of Barcelona Autonomous University, Spain, a pioneer in the field of the computer-aided detection and diagnosis of colorectal polyps, wrote the book Computer-Aided Analysis of Gastrointestinal Videos, which is the first comprehensive book in the world to compare and analyze different gastrointestinal image analysis systems. It aims to assist clinicians to complete key tasks such as lesion detection in colonoscopy images [16]. Barua et al. systematically searched the application of artificial intelligence in polyp detection in colonoscopy on MEDLINE, EMBASE and Cochrane Central. By calculating the relative risk, absolute risk and average difference of polyps, adenomas and colorectal cancer, the differences between colonoscopy and colonoscopy without AI were compared, summarized and analyzed. They found that an AI-based polyp detection system can effectively increase the detection rate of non-advanced adenomas and smaller polyps during colonoscopy [17].

However, in the process of polyp detection, the edge blur between adjacent tissues will be caused by the inherent characteristics of the colorectal image, insufficient brightness, noise, contrast and the technical limitations of the imaging equipment. In addition, because the features of the polyp image are composed of a large number of pixels, the traditional polyp detection methods do not preprocess the features of the original image, which may lead to the inability to obtain better features in the later feature extraction, resulting in the unsatisfactory detection effect in the later stage. In addition, in the process of colorectal image sample frame feature generation, it is often difficult to achieve the expected results with the traditional artificial image feature selection methods, and they cannot meet the practical application requirements in medical image processing and analysis. Therefore, to the process effectively extracting the global and local features of polyps and detecting targets urgently needs further research and exploration.

This paper discusses how to improve the recognition accuracy and efficiency of polyp detection from the perspective of artificial intelligence. This provides strong support for the missed diagnosis, early diagnosis and prevention of colorectal cancers in the process of polyp detection by clinicians. The main contributions of this article include: (1) In view of the lack of data, the Mosaic method is used in the data preprocessing stage to enhance the amount of training data in the data set; (2) CSPNet (Cross Stage Partial Networks) is used as the backbone network to extract the information features in the image, which solves the problem of gradient disappearance; and (3) The feature pyramid architecture with attention mechanisms is used to enhance the detection performance of varying-size polyps.

## 2. Related Works

### 2.1. Traditional Polyp Detection Algorithm

As we all know, the interpretation of endoscopic images is based on the experience of endoscopists and has a certain subjectivity, which makes it difficult for non-endoscopists or inexperienced endoscopists to make accurate judgments. Ideally, the real-time automatic polyp detection system can approach or even exceed the ability of the endoscopists, attract the eyes of the endoscopists to relevant lesions in real time, and help the endoscopists detect the presence of polyps and adenomas in a more reliable way. Computer-aided diagnosis (CAD) of colonoscopy has always been the focus of artificial intelligence research [18,19]. CAD can prompt the endoscopists to pay attention to the polyps that may be ignored through real-time display, improve the detection rate of adenomas and speed up the accurate optical biopsy characterization of colorectal polyps. If it is a non-neoplastic polyp, it can reduce unnecessary polypectomy.

Traditional polyp detection algorithms usually use artificial features, such as texture, shape and color features to detect polyps. Krishnan et al. used the curvature analysis method to identify the possible region where the polyp is located according to the curve direction and curvature [20]. Kang et al. divided the colonoscopy image into multiple regions and used watershed segmentation algorithm to carry out binary classification operations for each region, so as to judge whether there are polyps in that region [21]. Bernal et al. considered that the polyp surface has the property of three-dimensional protrusion, and they used the valley detection algorithm to detect the polyps [22]. According to the special texture features of polyp lesion areas, Wang et al. used G statistics and a neural network to judge the category of the enteroscopy image block [23]. Tjoa et al. used principal component analysis to reduce the dimension of the features in the texture spectrum and then used BP neural network to classify the features after dimension reduction [24]. Alexandre et al. first divided the polyp image into fixed size sub images, extracted the pixel values and coordinate values of the image, and used a support vector machine to classify the image [25]. Li et al. used a support vector machine to classify polyp images with different scales and then integrated multiple classifier results to judge whether it was a polyp [26].

However, because some polyps have a flat surface and similar shape and texture characteristics to the normal inner wall, it is easy to miss the detection of such polyps with the traditional algorithms; some of the inner walls of the colon have convex structures, so it is easy to mistakenly detect the inner wall of the colon as the polyps with the traditional algorithms. Therefore, the traditional polyp detection algorithm cannot complete the detection task well.

### 2.2. Polyp Detection Algorithm Based on Deep Learning

A convolutional neural network has been applied to the automatic detection of polyps under colonoscopy and achieved good results in improving the detection rate of polyps. However, the complex colonic environment leads to too many false positives, which will hinder the clinical application of the Cade system. Qadir et al. proposed an all-CNN real-time polyp detection model based on two-dimensional Gaussian shape prediction. The model is effective for flat and small polyps with unclear boundaries between the background and polyps [27]. TASHK et al. proposed a new method for polyp detection in colon capsule endoscopic images based on the new combination of RCNN and distance-regularized level set evolution. This method can not only reliably detect polyps from still images, but also predict and score the risk of malignant tumors [28]. Luo et al. proposed a high-performance, real-time automatic polyp detection system, which can improve the polyp detection rate in the actual clinical environment, especially on small polyps [29]. Yang et al. proposed a colon polyp detection and segmentation algorithm based on an improved mrcnn. The algorithm first trained large-scale data sets, extracted the initial model, and then retrained the small private data sets of patients [30]. In view of the large proportion of small polyps in heterogeneous data sets, in order to enhance the generalization ability of the model, Li et al. proposed a low-rank model by using the human resources network as the backbone to realize the accurate segmentation of polyps [31]. Wang et al. combined the classical vggnets and resnets models with the global average pooling and proposed two new lightweight network structures, vggnets gap and resnets gap, which not only had high classification accuracy, but also had fewer parameters [32]. Manouchehri et al. first proposed a new convolutional neural network for polyp frame detection based on the VGG network and then proposed a complete convolutional network and an effective post-processing algorithm for polyp segmentation [33]. Patel et al. compared a series of algorithms for polyp classification using CNN through self-built data sets and found that the performance of the vgg-19 was higher than those of the RESNET, densenet, senet and mnasnet [34].

In the preprocessing stage of polyp detection in colonoscopy image, a preprocessing method for automatic polyp detection based on a super-resolution convolutional neural network was proposed. Experiments show that this preprocessing method can achieve excellent performance in polyp localization even if the image resolution increases [35]. Tang et al. proposed a polyp detection method based on transfer learning technology for high-resolution colonoscopy images. The experimental results show that the polyp detection model can have high accuracy for polyp detection, but it had no obvious effect on polyp type classification [36]. Shen et al. proposed a transformer convolution network for end-to-end polyp detection. In this network, firstly, CNN was used for feature extraction, then the transformer encoder layer and convolutional layer were interleaved for feature coding and recalibration. The transformer decoder layer was used for object query, and finally, the feedforward network was used for target detection [37]. Liew et al. fused the improved depth residual network, principal component analysis and AdaBoost ensemble learning and proposed an automatic detection method of colon polyps based on depth residual network to classify endoscopic images. In addition, in order to minimize image interferences, the method used median filtering, image thresholding, contrast enhancement and normalization techniques to train the classification model [38]. Mulliqi et al. studied the importance of skip connection in the encoder–decoder structure of colorectal polyp detection. They found that with the improvement of the skip connection utilization, the segmentation results gradually improved, and the polyp segmentation architecture also achieved a better performance when the number of model parameters decreased significantly [39]. Mostafiz et al. first fused the color empirical mode decomposition features with the convolutional neural network features extracted from video frames and then classified the polyp images by support vector machine [40]. Hasan et al. extracted the features of the image through the PCA, removed the less important features and then diagnosed gastrointestinal polyps through the SVM and marked the detected polyp regions [41].

In order to improve the detection efficiency, some target detection algorithms based on the yolo series have been proposed [42]. Guo et al. proposed an automatic polyp detection algorithm based on yolov3 structure and active learning, which can effectively reduce the false positive rate in polyp detection [43]. Cao et al. designed a feature extraction and fusion module and combined it with the yolov3 network. This method can integrate the semantic information of a high-level feature map and low-level feature map and is superior to other methods in the detection of small polyps [44]. Pacal et al. proposed a real-time automatic polyp detection method based on Yolov4. They applied the cspnet network to the whole architecture and added mish activation function, Diou loss function and transformer block to the architecture. This method has higher accuracy and performance than previous methods [45].

However, with the increasing resolution of the polyp image during colonoscopy, the feature of polyp image is composed of a large number of pixels. The traditional polyp detection methods do not effectively preprocess the features of the original image. In the process of polyp detection, due to the inherent characteristics of colorectal images, insufficient brightness, noise, contrast and technical limitations of the imaging equipment, and the edge between adjacent tissues can be blurred, which may lead to the inability to obtain better features in future feature extraction. In addition, the requirements for real-time polyp detection rate are becoming higher and higher. Although the automatic polyp detection system has been comprehensively studied in the past decade, there is still a lack of evidence about the ability of this technology to locate and track polyps in the process of real-time colonoscopy in clinical practice.

## 3. Materials and Methods

### 3.1. Network Configuration

The proposed method was trained by stochastic gradient descent (SGD) and backpropagation in an end-to-end way on a cloud-computing platform configured with eight 16 GB GPUs, a 16-core CPU, and a 64 GB memory.

### 3.2. Dataset

**Figure 1:**
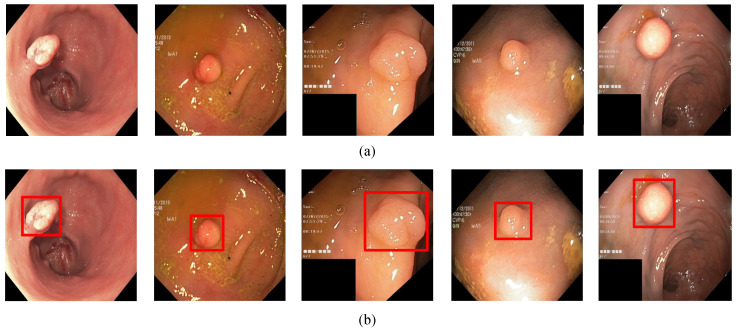
The polyp object detection Kvasir-SEG dataset: (**a**) Polyp image samples, (**b**) ground-truths with bounding boxes.The red squares in the figure are the bounding boxes of the polyps.

**Figure 2:**
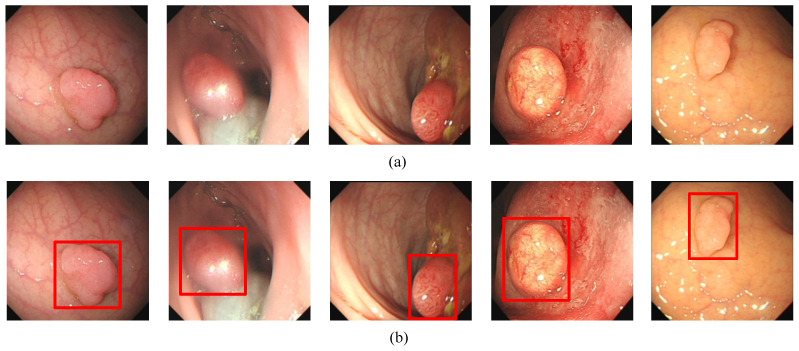
The polyp object detection WCY dataset: (**a**) Polyp image samples, (**b**) ground-truths with bounding boxes. The red squares in the figure are the bounding boxes of the polyps.

In order to evaluate the algorithm, we used the Kvasir-SEG data set, which is the first multi-class data set for gastrointestinal (GI) disease detection and classification and contains a total of 1000 pictures. In addition, we collected 1000 pictures from the endoscopy center of the local hospital, each with 1–3 colon polyps, and constructed the WCY data set. During the experiment, we used the five-fold cross-validation method to divide the data set. Each time 800 pictures were randomly selected for training, and 200 pictures were used for testing. Figure 1a and Figure 2a are sample examples in the two different data sets, and Figure 1b and Figure 2b are the cases of using bounding boxes to label two different data. Among them, when labeling the data set, we used the Labelme toolkit for labeling.

In this paper, YOLOv5 based on an attention mechanism was mainly used for the target detection of polyp images. This method uses the idea of regression, using the entire image as the input of the network and directly returning the target frame of this position in multiple positions of the image. It is mainly divided into five parts: Input, backbone network, neck, attention mechanism and prediction. 

### 3.3. Input

**Figure 3:**
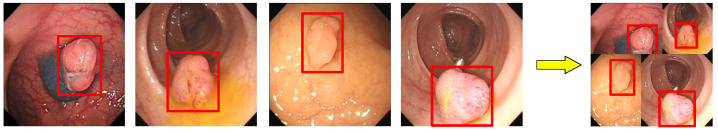
Mosaic data enhancement diagram. The red squares in the figure are the bounding boxes of the polyps.

In the data preprocessing stage, due to the lack of polyp data, YOLOv5 uses Mosaic data enhancement at the input to enhance the amount of training data in the two data sets. Mosaic first reads four pictures and then performs operations such as flipping, zooming and color gamut changes on the four pictures. Finally, they are placed in the four directions, and the pictures and the frame are combined to form a new picture. Additionally, it obtains the frame corresponding to this new picture. During the splicing process, each of the four images are covered by the rest of the images, or the label frame in the image will be blocked or covered by several other images. If the picture box appears beyond the edges of the two pictures, we need to delete it. The mosaic data enhancement schematic is shown in Figure 3.

In addition, in network training, YOLOv5 outputs the prediction frame based on the initial anchor frame and then compares it with the ground-truth of the real frame, calculates the gap between these two and then updates it in the reverse direction to adaptively calculate the best anchor frame in different training sets.

### 3.4. Backbone

When the artificial neural network is trained in back propagation, as the number of hidden layers increases, when calculating the gradient of the loss function to the weight, the gradient becomes smaller and smaller as it propagates backward. This means that the neurons in the front layers of the network are much slower than those trained later and will not even change. In some cases, the gradient values almost disappear, that is, the phenomenon of gradient disappearance. 

YOLOv5 uses CSPNet (Cross Stage Partial Network) as the backbone network to extract information features in the images. The backbone network replicates the feature map of the base layer and uses a dense block to transfer the copied feature map to the next stage, thereby separating the feature map of the base layer. This can effectively alleviate the problem of gradient disappearance, support feature propagation and encourage the network to reuse features, thereby reducing the number of network parameters. CSPNet solves the problem of repeating the gradient information of network optimization in the backbone network of other large-scale convolutional neural network frameworks and integrates the changes of gradients into the feature map from the beginning to the end, thus reducing the amount of model parameters and FLOPS (floating point operations per second), which can ensure accuracy while reducing the amount of calculations. 

### 3.5. Neck

**Figure 4:**
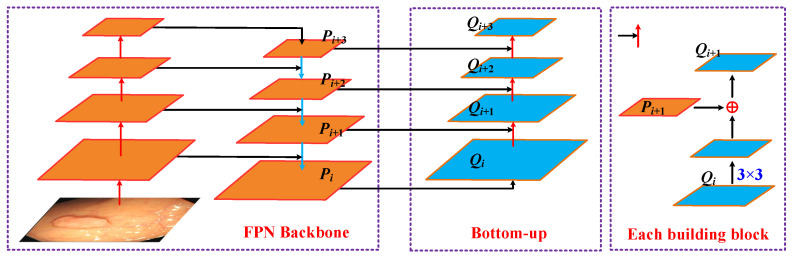
Illustration of Path aggregation network.

YOLOv5 uses SPP (spatial pyramid pooling) to enhance the model’s detection of objects with different scales and uses PANET (Path aggregation network) as the neck for feature aggregation [46]. The feature extractor of the path aggregation network adopts a new FPN (Feature Pyramid Networks) structure that enhances the bottom-up path, which improves the propagation of low-level features. Each stage of the third path takes the feature maps of the previous stage as the input and processes them with a 3 × 3 convolutional layer. The output is added to the feature map of the same stage of the top-down path through the horizontal connection, and these feature maps provide information for the next stage. An illustration of the Path aggregation network is as follows:

In this framework, *P_i_*, *P_i_*_+1_, *P_i_*_+2_ and *P_i_*_+3_ are the feature levels generated by the FPN. The enhancement path gradually approaches the top *P_i_*_+3_ from the lowest layer *P_i_*, and the space size is gradually down-sampled by a factor of 2. In addition, in the bottom-up process, *Q_i_*, *Q_i_*_+1_, *Q_i_*_+2_ and *Q_i_*_+3_ are the newly generated feature maps for *P_i_* and *P_i_*_+3_, respectively. The detailed process is shown in Figure 4: First, each feature map *Q_i_* uses a 3 × 3 convolutional layer with a step size of 2 to reduce the space size. Then, each element of the feature map *P_i_*_+1_ is added to the down-sampling map through the horizontal connection. The fused feature map is processed by another 3 × 3 convolutional layer to generate *Q_i_*_+1_. This is an iterative process that ends after generating *Q_i_*_+3_.

At last, adaptive feature pooling is used to restore the damaged information path between each candidate area and all feature levels and aggregate each candidate area on each feature level to avoid arbitrary allocation. Through this step, polyps of different sizes and scales can be identified.

### 3.6. Attention Mechanism

**Figure 5:**
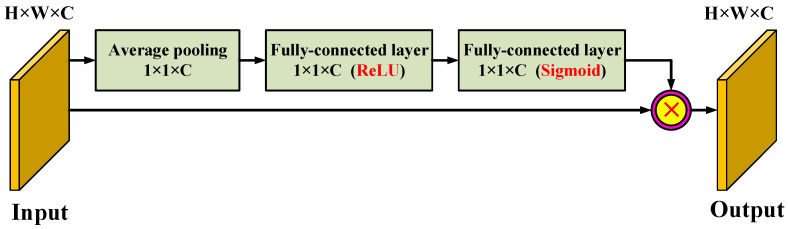
The architecture of the attention mechanism module.

In the learning process of the model, the more parameters the model has, the richer the amount of information stored, but it will bring about the problem of information overload. To alleviate this issue, we integrated a self-attention module on the top layer of each stage of the feature extraction backbone network. The model in this paper first obtains important candidate target regions by scanning the global image and then strengthens the contribution of the information-rich feature channel through the increased attention mechanism and weakens the interference of useless channels. Through this mechanism, limited attention resources can be used to quickly filter out high-value information from a large amount of information. The architecture of the attention mechanism module is shown in Figure 5.

For the input of the attention module, first, we performed a global average pooling operation on the channel and converted the input feature map into a channel descriptor. In this way, the statistical characteristics of the channel could be obtained from a global perspective. Then, we followed the two fully connected layers to further explore the dependencies between the channels. Specifically, the two fully connected layers are activated by the rectified linear unit (ReLU) and the sigmoid function, respectively. The second probabilistic encoding fully connected layer constitutes an attention descriptor, and the elements of the attention descriptor reflect the amount of information and saliency of the corresponding channels of the input feature map. This attention descriptor acts as a weight adjuster to recalibrate the input feature map. Finally, by multiplying the attention descriptor with the input feature map, an information feature emphasizing feature map was generated. Finally, we superimposed global features and local features and fused the superimposed features through a 1 × 1 convolutional layer to complete the colorectal image sample frame feature generation model and used the final generated depth features as the input part of the target detection model.

### 3.7. Prediction

In the YOLOv5 model, the head model is the same as the previous Yolov3 and Yolov4, which is mainly used in the final inspection part. It applies anchor boxes to the feature map and generates the final output vector with class probabilities, object scores and bounding boxes.

The choice of activation function is crucial for deep learning networks. In YOLOv5, the Leaky ReLU activation function is used in the middle/hidden layer, and the Sigmoid activation function is used in the final detection layer.

The Leaky ReLU activation function is as follows:

(1) $$y_{i}=\left\{ \begin{array}{l} x_{i}\begin{array}{cc} & x_{i}\geq 0 \end{array}\\ \frac{x_{i}}{a_{i}}\begin{array}{cc} & x_{i}<0 \end{array} \end{array}\right.$$

in which $a_{i}\in (1,+\infty )$.

The Sigmoid activation function is as follows:

(2) $$f(z)=\frac{1}{1+e^{-z}}$$

The loss function is an important indicator to measure the generalization ability of the model. We trained this model by calculating the gap between the predicted value and the true value of the data. The ultimate goal of optimizing the model was to reduce the loss value as much as possible without fitting. YOLOv5 uses the following *GIOU_Loss* as the loss function of the Bounding box.

(3) $$IoU=\frac{\left|A\cap B\right|}{\left|A\cup B\right|}$$

(4) $$GIoU=IoU-\frac{\left|C\backslash (A\cup B)\right|}{\left|C\right|}$$

(5) GIoU_Loss=1−GIoU

Among them, *A* and *B* are two arbitrary boxes, and *C* is the smallest closed shape containing the two boxes *A* and *B*.

After calculating the loss value of the model, the next step was to use the loss value to optimize the model parameters. YOLOv5 uses SGD as the optimization function by default, but if the training set is small, then Adam (A method for stochastic optimization) is selected as the optimization function. Adam is a first-order optimization algorithm that can replace the traditional stochastic gradient descent process. It can iteratively update neural network weights based on the training data. It requires less memory and is suitable for solving problems containing very high noise or sparse gradients. The hyper-parameters can be explained intuitively, and only a small amount of parameter adjustment is required. Its calculation process is as follows:

Step 1: Initialization $V_{\alpha w}=0,S_{\alpha w}=0,V_{\alpha b}=0,S_{\alpha b}=0$.

Step 2: In the *t*th iteration, use the mini-batch gradient descent method to calculate *dw* and *db.*

Step 3: Calculate the weighted average of Momentum index.

(6) $$V_{\alpha w}=\beta_{1}V_{\alpha w}+(1-\beta_{1})dw,\;V_{\alpha b}=\beta_{1}V_{\alpha b}+(1-\beta_{1})db$$

Step 4: Update with RMSprop.

(7) $$S_{\alpha w}=\beta_{1}S_{\alpha w}+(1-\beta_{2})dw^{2},\;S_{\alpha b}=\beta_{2}S_{\alpha b}+(1-\beta_{2})db$$

Step 5: Calculate the deviation correction of Momentum and RMSprop.

(8) $$V_{dw}^{correct}=V_{dw}/(1-\beta_{1}{}^{t}),\;V_{db}^{correct}=V_{db}/(1-\beta_{1}{}^{t})$$

(9) $$V_{dw}^{correct}=S_{dw}/(1-\beta_{2}{}^{t}),\;S_{db}^{correct}=S_{db}/(1-\beta_{2}{}^{t})$$

Step 6: Update weight.

(10) $$w=w-\alpha \frac{v_{dw}^{correct}}{\sqrt{s_{dw}^{correct}}+\varepsilon },\;b=b-\alpha \frac{v_{db}^{correct}}{\sqrt{s_{db}^{correct}}+\varepsilon }$$

Among them, *α* is the learning rate or step size factor that controls the update rate of the weights. A larger value of *α* will result in faster initial learning before the learning rate is updated, and a smaller value of *α* will make the training converge to better performance. *β*_1_ and *β*_2_ are the exponential decay rates of the first and second moment estimates, respectively. In order to avoid the denominator being 0, *ε* is a non-zero number.

## 4. Results

### 4.1. Polyp Object Detection

In order to evaluate the performance of the algorithm for detecting polyps, this paper uses *precision*, *recall*, *F-score* and detection time as four indicators to measure the performance of the algorithm. The formulas are as follows:

(11) $$precision=\frac{TP}{TP+FP}$$

(12) $$recall=\frac{TP}{TP+FN}$$

(13) $$F-score=2\times \frac{precision\times recall}{precision+recall}$$

Among them, *TP* is the number of true positives, that is, the number of correctly detected and labeled polyp instances. *FN* is the number of false negatives, that is, the number of polyps that have not been correctly detected. *FP* is the number of false positives, that is, the number of polyps that are not polyps. Therefore, *precision* measures the proportion of correctly labeled polyps in all pictures predicted to be polyps and *recall* measures the proportion of polyps detected in all polyp images. *F-Score* is the harmonic average of *precision* and *recall* that provides an overall evaluation.

**Figure 6:**
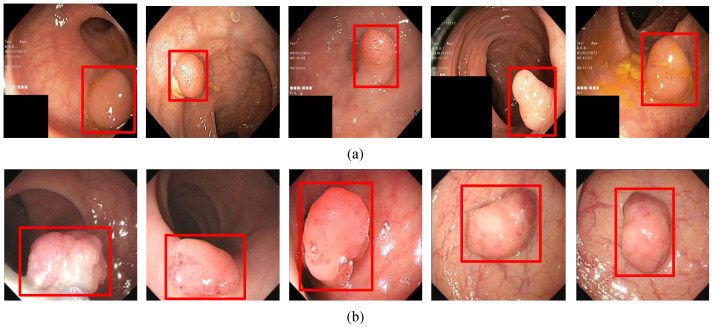
(**a**) A subset of the detection results of single polyp in Kvasir-SEG data set. (**b**) A subset of the detection results of single polyp in WCY data set. The red squares in the figure are the bounding boxes of the polyps.

**Figure 7:**
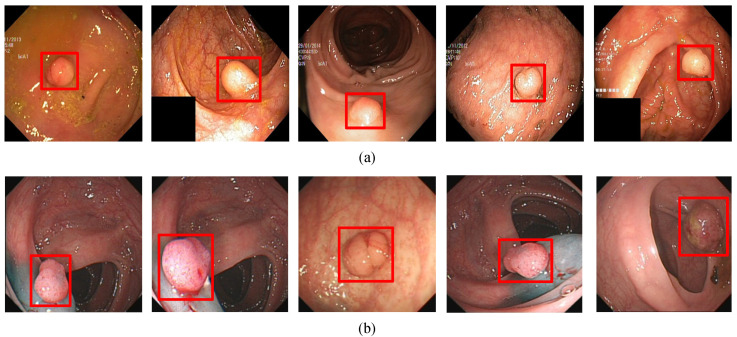
(**a**) A subset of the detection results of small target polyps in Kvasir-SEG data set. (**b**) A subset of the detection results of small target polyps in WCY data set. The red squares in the figure are the bounding boxes of the polyps.

**Figure 8:**
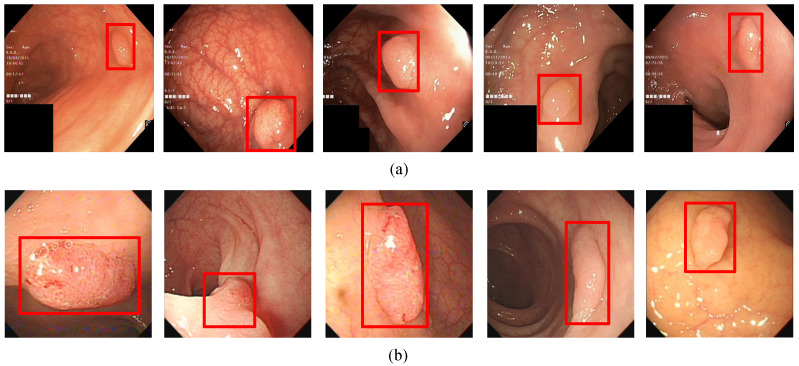
(**a**) A subset of the detection results of multiple target polyps showing low contrasts to the background in Kvasir-SEG data set. (**b**) A subset of the detection results of multiple target polyps showing low contrasts to the background in WCY data set. The red squares in the figure are the bounding boxes of the polyps.

**Figure 9:**
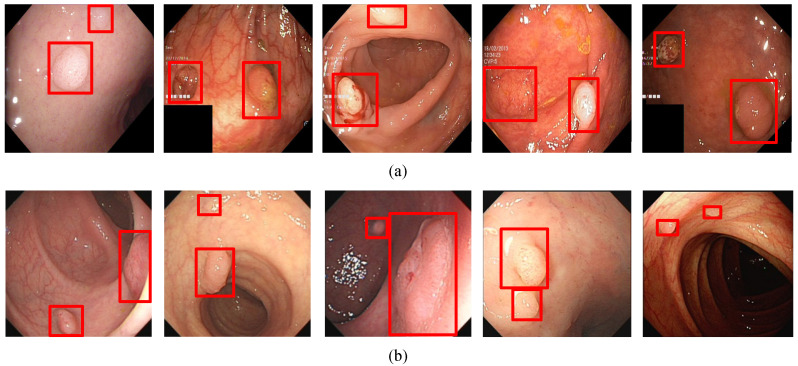
(**a**) A subset of the detection results of multiple target polyps in Kvasir-SEG data set. (**b**) A subset of the detection results of multiple target polyps in WCY data set. The red squares in the figure are the bounding boxes of the polyps.

In order to visualize the detection results, Figure 6, Figure 7, Figure 8 and Figure 9 show the detection effects of polyps in different data sets.

### 4.2. Comparisons with State-of-the-Art Methods

In order to further evaluate the performance of the proposed polyp detection method, we compared the algorithm proposed in this paper with some classic commonly used models and analyzed the findings. Among them, CNN, R-CNN and the Faster R-CNN method use a two-stage framework detection model. They first extract candidate regions of polyp images and then classify the candidate regions with a deep learning method. Yolov4 is a regression method based on deep learning, which belongs to the detection model of a one-stage framework. Table 1 shows the evaluation results of different algorithms in the two data sets.

## 5. Discussion

In addition, in the polyp detection process, it may appear that the color of the polyp and the background picture are similar. For handling this type of polyp pictures, the experimental results are shown in Figure 8. It can be seen from the figure that in this category of images, the color of the polyp is close to the background color, and the detection algorithm in this article can accurately detect it.

Since, in polyp detection, there may be multiple polyps in an image, the algorithm in this paper also achieved better results in this case. The experimental results are shown in Figure 9. It can be seen from the figure that the method in this paper can detect polyps of different sizes in a picture at the same time. Even if multiple polyps of small sizes appear in a picture, the algorithm can accurately detect them. Therefore, in general, the algorithm in this paper can achieve a promising performance no matter what type of polyp picture is used.

As can be seen from Table 1, our method achieved excellent performance on the test set. In the Kvasir-SEG data set, the *precision* was 0.915, the *recall* rate was 0.899 and the *F-score* was 0.907. In the WCY data set, the *precision* was 0.913, the *recall* was 0.921 and the *F-score* was 0.917. Specifically, this method uses full-image information when predicting the target window using each network, which greatly reduces the false positive rate. Compared with the one-stage deep learning model YOLO-v4, the overall performance of the *F-score* on the two data sets improved by about 0.026 and 0.032, respectively. Compared with the CNN, the overall performance of the *F-score* on the two data sets improved by about 0.032 and 0.019, respectively. This is mainly because the resolution of the polyp image is very large, and there are many small targets in it that need to be detected. If it is directly input to the detection network, the detection effect is not good enough. Although the precision of Faster R-CNN algorithm is higher than our method, the recall and *F-score* are lower than our method. Because there are multiple polyps in WCY dataset and the contrast between polyps and background is not strong, the Faster R-CNN algorithm will miss labeling. The method in this paper adopts the Mosaic data enhancement method at the input end and performs splicing through random scaling and other methods. This has a better detection effect on small targets, so the effect is better. 

Furthermore, in the fields of polyp detection and cancer detection, we need to improve the recall rate as much as possible, reduce the false negative rate and avoid missed detection by ensuring the accuracy rate.

The training and prediction time of the model are other important evaluation criteria to measure the performance of the algorithm. If the time complexity is too high, it leads to a long duration of model training and prediction, which cannot quickly verify the idea and improve the model and cannot achieve fast prediction. 

It can be seen from Table 1, the polyp detection process of the R-CNN algorithm takes a long time, and each photo takes about one second. A series of methods such as CNN, RCNN, etc., first use the Selective Search algorithm by inputting image attribute information to generate more reliable candidate regions on different color patches and then use deep learning. The method of extracting features and classifying these regions can solve the problems caused by sliding windows in traditional detection methods. However, in actual applications, each picture will have two thousand candidate frames. CNN feature extraction is performed on each candidate frame, and then classification and regression are performed. The amount of calculation is large, and the feature takes up a large amount of memory space and overlaps. There will be a lot of repeated calculations in the convolution operation, and the entire process requires a lot of time. These algorithms cannot meet the real-time requirements in terms of speed. 

Although the running speed of Faster RCNN has been greatly improved in traditional methods, and it can process three pictures in about one second, Yolo series algorithms convert the polyp detection problem into a single regression problem of directly extracting bounding boxes and category probability from images. Yolo-v4 and other regression methods based on deep learning, using the idea of regression, use the entire image as the input to the network and directly return the target frame of this position in multiple positions of the image and the category to which the target belongs, greatly speeding up the speed of detection. 

In contrast, the improved algorithm based on Yolov5 proposed in this paper is closer to the two-level target detection algorithm in accuracy. In terms of detection time, it only takes one-tenth of the Faster R-CNN algorithm to complete the detection of a picture, and it only takes about 30 milliseconds to process a picture. Therefore, YOLOv5 algorithm based on attention mechanism has a very fast detection speed while ensuring accuracy. 

## 6. Conclusions

At present, artificial intelligence polyp detection technology is still in its infancy. Compared with traditional statistics or expert systems, deep learning methods usually improve the performance and detection accuracy of most image target detection. In response to this problem, this article focuses on the fusion of the attention mechanism and YOLOv5 for polyp target detection. On the basis of ensuring high detection accuracy, the detection time was greatly improved, especially for the small targets and the polyp images with weak contrasts. Therefore, the model designed in this article has a certain degree of innovation and application value, and also has a certain guiding role for the clinical work for the endoscopists. However, there are still some challenges in the application of artificial intelligence in polyp detection. For example, the tagger of polyp image needs to have a certain medical background, and the cost of obtaining high-quality medical image tagging is even higher than that of obtaining medical images; there is a large gap in the amount of data between different lesion types and normal medical images. Establishing an excellent training data set is very important. In the future, we will carry out further research around the existing problems.

## Figures and Tables

**Table 1:** Performance of polyp detection between different algorithms. In the table, we bold the optimal results obtained by each index.

	Kvasir-SEG Dataset				WCY Dataset			
Methods	*Precision*	*Recall*	*F-score*	Time(s)	*Precision*	*Recall*	*F-score*	Time(s)
CNN	0.879	0.871	0.875	1.861	0.908	0.889	0.898	2.176
R-CNN	0.910	0.887	0.898	1.175	0.911	0.892	0.901	1.298
Faster R-CNN	0.914	0.896	0.905	0.382	**0.916**	0.897	0.906	0.374
Yolov4	0.883	0.880	0.881	0.032	0.895	0.876	0.885	0.037
Ours	**0.915**	**0.899**	**0.907**	**0.028**	0.913	**0.921**	**0.917**	**0.030**

## Data Availability

Search results are available from the authors.
